# Bullous Pemphigoid IgG Induces Cell Dysfunction and Enhances the Motility of Epidermal Keratinocytes via Rac1/Proteasome Activation

**DOI:** 10.3389/fimmu.2019.00200

**Published:** 2019-02-12

**Authors:** Duerna Tie, Xia Da, Ken Natsuga, Nanako Yamada, Osamu Yamamoto, Eishin Morita

**Affiliations:** ^1^Department of Dermatology, Shimane University Faculty of Medicine, Izumo, Japan; ^2^Department of Dermatology, Hokkaido University Graduate School of Medicine, Sapporo, Japan; ^3^Division of Dermatology, Department of Medicine of Sensory and Motor Organs, Faculty of Medicine, Tottori University, Yonago, Japan

**Keywords:** bullous pemphigoid, keratinocyte, IgG, cell adhesion, cell migration

## Abstract

Bullous pemphigoid (BP) is an autoimmune disease characterized by the formation of blisters, in which autoantibodies mainly target type XVII collagen (ColXVII) expressed in basal keratinocytes. BP IgG is known to induce the internalization of ColXVII from the plasma membrane of keratinocytes through macropinocytosis. However, the cellular dynamics following ColXVII internalization have not been completely elucidated. BP IgG exerts a precise effect on cultured keratinocytes, and the morphological/functional changes in BP IgG-stimulated cells lead to the subepidermal blistering associated with BP pathogenesis. Based on the electron microscopy examination, BP IgG-stimulated cells exhibit alterations in the cell membrane structure and the accumulation of intracellular vesicles. These morphological changes in the BP IgG-stimulated cells are accompanied by dysfunctional mitochondria, increased production of reactive oxygen species, increased motility, and detachment. BP IgG triggers the cascade leading to metabolic impairments and stimulates cell migration in the treated keratinocytes. These cellular alterations are reversed by pharmacological inhibitors of Rac1 or the proteasome pathway, suggesting that Rac1 and proteasome activation are involved in the effects of BP IgG on cultured keratinocytes. Our study highlights the role of keratinocyte kinetics in the direct functions of IgG in patients with BP.

## Introduction

Bullous pemphigoid (BP) is a skin-specific autoimmune disease characterized by subepidermal blisters (1–4). Systemic glucocorticoids and immunosuppressive agents are effective treatment options for BP (5, 6), but they increase the risk of lethal infections, particularly in elderly patients (7, 8). The autoimmune mechanisms involved in BP have been well-discussed based on clinical and experiential evidence (9, 10). IgG is the principal antibody involved, which recognizes the non-collagenous 16a domain (NC16a) of type XVII collagen (ColXVII, also known as BP180 or BPAg2) (9, 11–14).

ColXVII, a transmembrane protein, is located either at the core of the hemidesmosome in the skin (1) or in the hemidesmosome protein complexes of cultured keratinocytes (15, 16). ColXVII contains a long extracellular collagenous tail, which traverses the basement membrane zone (BMZ) (17), and serves as a signaling connector and/or a “mucilage” between the epidermis and the dermis (18–21). A genetic deficiency in ColXVII leads to junctional epidermolysis bullosa, resulting in the separation of the epidermis from the dermis (22, 23). Binding of the specific IgG to ColXVII is considered to contribute to intra-lamina lucida blistering in subjects with BP (24–26).

ColXVII-NC16a is a juxtamembrane region that plays a central role in the formation of the collagen-like-triple helix (27, 28). Moreover, IgG binding to ColXVII-NC16a is the most essential initial event in BP, and detection of the ColXVII-NC16a-specific IgG is important for diagnosing BP (10, 29, 30). The pathological progress of blistering after BP IgG binding to ColXVII-NC16a remains a debatable issue. Previously, complement activation was considered a major cause of blistering, because immunofluorescence studies of skin biopsies from patients with BP have shown that most patients with BP exhibit C3 deposition. Moreover, according to laboratory investigations, the binding of IgG to murine ColXVII triggers complement activation, mast cell degranulation, and neutrophil infiltration, suggesting that the formation of blistering lesions require both IgG and the recruitment and activation of complement (31–35). Recently, another BP pathomechanism has been advocated in which IgG exerts a direct effect on inducing blistering in patients with BP (36, 37). Evidence supporting these direct effects of BP IgG has been reported. Immunofluorescence studies have revealed that >10% BP cases are positive for BP IgG but negative for C3 deposition (31, 35). Based on the findings from some case reports, IgG staining shows an intercellular pattern in basal cells rather than a linear deposition along the BMZ (38). Clinical observations identified a non-inflammatory BP variant that is often associated with dipeptidyl peptidase-4 inhibitors application (39). BP IgG has been reported to directly induce blisters between the epidermis and the dermis without complement activation (40, 41), influence cell morphology (42), and “deplete” ColXVII in cell culture systems (37). The binding of BP IgG to ColXVII has also been reported to cause internalization of the immune complex by forming macropinosomes, resulting in decreased cell adhesion at the single cell level (43).

Although our understanding of the mechanism by which BP IgG induces blistering at the single-cell-level has improved, knowledge regarding macropinosome formation in the pathogenesis of BP is still lacking. A macropinosome is a large endocytic vacuole formed during macropinocytosis (44). Macropinosome formation acts as an entry site for intracellular pathogens (e.g., bacteria) that helps cells recognize the antigens/microbe-associated molecules (45, 46) and accumulate the metabolites required for proliferation (e.g., cancer cells) (47). However, hyperstimulation of macropinosomes leads to cell death (48, 49). Thus far, the changes in the morphology of keratinocytes during BP IgG-induced macropinosome formation are incompletely understood. We hypothesized that BP IgG contributes to the pathogenesis of BP by inducing keratinocyte dysfunction. The aim of this study was to clarify the morphological and functional changes in human keratinocytes incubated with BP IgG and the subsequent effects of inhibitors of macropinosome formation on these morphological and functional events.

## Materials and Methods

### Cell Culture

Normal human epidermal keratinocytes (NHEKs) were obtained from the American Type Culture Collection (ATCC, Manassas, VA, USA) and cultured in serum-free media (with calcium chloride at a final concentration of 0.09 mM, Defined Keratinocyte SFM supplemented with growth factors, Gibco, Invitrogen Corporation, Carlsbad, CA, USA), 1% penicillin-streptomycin (Wako Pure Chemical Industries, Japan), and 25 ng/ml amphotericin B (Wako Pure Chemical Industries) at 37°C in a humidified atmosphere containing 5% CO_2_. Cells were used after two to six passages in all experiments.

### Antibody Purification

Plasma samples were obtained from three patients with BP (BP-1, BP-2, and BP-3), and eluents were obtained by a double filtration plasmapheresis treatment. BP IgGs fractions were isolated from plasma using a HiTrap Protein G HP column (GE Healthcare, Marlborough, MA, USA) with a fast protein liquid chromatography system (Amersham Biosciences, Marlborough, MA, USA), according to the manufacturer's protocol. The functions of BP IgGs were confirmed using indirect immunofluorescence staining of the salt-spilt human skin and a ColXVII-NC16a chemiluminescent enzyme immunoassay test (CLEIA, SRL Inc., Hachioji, Japan). Immunoblotting using the normal human epidermal extracts confirmed that the BP IgGs used in this study only reacted with ColXVII (180 kDa) (data not shown) and not with other antigens. The purified BP IgGs were concentrated through extensive washed with 0.9% NaCl and 50 K ultrafiltration (Millipore, Lexington, MA), and then filter-sterilized (pore size 0.22 μm; Millipore). The pooled IgGs from healthy people (normal IgG, Kenketsu Glovenin I, Nihon Pharmaceutical Co. Ltd., Tokyo, Japan) were dissolved in 0.9% NaCl to 50 mg/ml. Normal rabbit IgGs were purified using a Pierce classic IP kit (Thermo Fisher Scientific, MA, USA) with normal rabbit serum (ab7487, Abcam, Cambridge, UK). All IgGs protein concentrations were measured spectrophotometrically at 280 nm and samples were stored at −20°C.

This study was approved by the Ethics Committee of Shimane University and the Dean of the Faculty of Medicine (approval nos. 1746 and 2679).

### Reagents

Carbobenzoxy-Leu-Leu-leucinal (MG132), cytochalasin D, and n6-[2-(4-diethylamino-1-methyl-butylamino)-6-methyl-pyrimidin-4-yl]-2-methyl-quinoline-4,6-diamine trihydrochloride (NSC23766) were purchased from Sigma (St. Louis, MO, USA). MG132 and cytochalasin D were dissolved in DMSO to generate 10 and 2 mM stock solutions, respectively. NSC23766 was dissolved in distilled water to generate a 10 mM stock solution. All inhibitor stock solutions were frozen at −20°C. For the application of inhibitors to cell cultures, all reagents were diluted in warm culture medium at the indicated concentrations. NHEKs were pretreated with cytochalasin D for 30 min, NSC23766 for 1 h, or MG132 for 0 h, and then IgGs were added prior to the analysis.

### Flow Cytometry Analyses

Upon reaching ~60% confluence, NHEKs were treated with 2 mg/ml IgGs obtained from patients with BP (BP IgGs) or normal IgG. After 0, 0.25, 0.5, 1, 2, and 6 h incubations, the cells were washed with Dulbecco's phosphate-buffered saline (DPBS). Non-trypsin detached cells were immediately fixed with 4% paraformaldehyde and resuspended in DPBS. Cells were first incubated with a monoclonal rabbit IgG specific for human ColXVII-COOH (ColXVII IgG, ab184996, Abcam) and then incubated with anti-rabbit IgG-Alexa488 (ab150077, Abcam) to analyze the cell surface ColXVII expression. Cells were directly incubated with anti-human IgG-FITC (Dako, Copenhagen, Denmark) and examined using flow cytometry to determine the amount of IgG bound to the cell surface.

### Immunostaining Assay

NHEKs were seeded on sterile glass coverslips at a density of 0.2 × 10^6^ cells/ml, and after a 16 h incubation, NHEKs were pretreated with or without cytochalasin D, NSC23766, or MG132, and incubated with BP IgGs (2 mg/ml), normal IgG (2 mg/ml), or ColXVII IgG (12 μg/ml) for 0, 0.5, or 2 h. Afterwards, the cells were washed with DPBS, fixed with cold 4% (w/v) paraformaldehyde for 10 min, permeabilized and then blocked with 0.3 M (w/v) glycine/1% (w/v) BSA/10% (v/v) normal rabbit serum/0.1% (v/v) Tween-20 in DPBS for 1 h at room temperature (RT). NHEKs that had been treated with IgGs for 0 h were further incubated with BP IgGs (2 mg/ml) or normal IgG (2 mg/ml) for 1 h at RT or incubated with ColXVII IgG (12 μg/ml) overnight at 4°C. Cells were then washed three times with DPBS. All cells treated with IgGs were subsequently incubated with Alexa488- or FITC-conjugated secondary antibodies and rabbit or human IgG for 1 h at RT. The nuclei were stained with 4′, 6-diamidino-2-phenylindole (DAPI, Thermo Fisher Scientific). Cells on the coverslips were washed, mounted in mounting medium (Vector Laboratories, Burlingame, CA, USA), and viewed under a confocal scanning laser microscope (FV-100V, Olympus, Tokyo, Japan).

For the 3D reconstruction assay, a series of z-planes captured at 0.38 μm intervals were imaged with an Olympus FV-100V confocal microscope after staining. The 2D z-stack images from each channel were projected onto one 3D plane using the Olympus FV-100V software 3D view function. A red box indicated projected z-stack images, a yellow boxed region indicated one frame of a z-stack image, and a cut-off XZ or XY-section was shown in a yellow slice to better display the staining.

Quantification of the fluorescence intensity was performed using ICY software (icy.bioimageanalysis.org).

### Cell Counting Assay

NHEKs were cultured in 96-well culture plates to 40–60% confluence in the presence or absence of cytochalasin D, NSC23766, or MG132. Cells were labeled with Hoechst 33342 (Thermo Fisher Scientific) and treated with BP IgGs (2 mg/ml), normal IgG (2 mg/ml), ColXVII IgG (12 μg/ml), or normal rabbit IgG (2 mg/ml). Live cell imaging was performed with an In Cell analyzer 2000 (GE Biosciences, Piscataway, NJ, USA) equipped with a ×20 objective. After a 6 h incubation with IgGs, the numbers of adherent cells in a ×20 area (0.57 mm^2^) were automatically counted every hour with the In Cell Analyzer 1000 Workstation software (GE Biosciences) using the cell count analysis module.

### Transmission Electron Microscopy (TEM) and Scanning Electron Microscopy (SEM)

NHEKs were incubated with BP IgGs (2 mg/ml) or normal IgG (2 mg/ml). Cells grown on four-well culture slides to 60% confluence were observed with TEM and the cells grown on 15 mm round cover-slips to 60% confluence were observed with SEM. For TEM, after the incubation with the indicated IgGs, cells were washed and immediately fixed with 2.5% (v/v) glutaraldehyde (Sigma) in 0.1 M cacodylate buffer (pH 7.4, Wako Pure Chemical Industries) for 2 h. Cells were then washed and postfixed with 1% (v/v) osmium tetroxide for 1 h and 1% (v/v) uranyl acetate for 1 h at 4°C in the dark. Dehydration was achieved in a graded series of ethanol solutions. Dehydrated cells were cleared in n-butyl glycidyl ether (QY-1), embedded in Epon (TAAB Epon 812 Resin, Berkshire, England), and cut into ultrathin sections at a thickness of 80 nm using an ultramicrotome. Sections were collected on a 300-mesh copper grid, and ultrathin sections were double stained with uranyl acetate and lead citrate. For SEM, after the incubation with the indicated IgGs, the debris were removed from the culture by gentle washes with sterile DPBS. Cells were fixed with 2.5% (v/v) glutaraldehyde for 2 h at 4°C and washed with a 0.1 M cacodylate buffer (pH 7.2) solution. Cells were postfixed with 1% (v/v) osmium tetroxide for 1 h at 4°C, stained with 1% (w/v) tannic acid for 1 h at 4°C in the dark, and then fixed with 1% (v/v) osmium tetroxide for 1 h at 4°C. Cells were subsequently dehydrated in an increasing gradient of ethanol solutions, critical point dried in liquid CO_2_, mounted on aluminum stubs with the cell layer facing up, and then coated with gold. Cells were observed with a JSM-6510 scanning electron microscope (JEOL Co., Ltd., Tokyo, Japan) at a 10 kV accelerating voltage.

### LysoTracker® Green DND-26 Staining Assay

NHEKs seeded on a 35 mm dish (Ibidi GmbH, Martinsried, Germany) at ~40% confluence were treated with BP IgGs (2 mg/ml) or normal IgG (2 mg/ml) for 2 h. Cells were stained with 50 nM Lysotracker® Green DND-26 (Thermo Fisher Scientific) for 15 min in the dark and the fluorescence of lysosomes was immediately detected using confocal microscopy (Olympus FV-1000). Images were quantified using CellProfiler image analysis software (cellprofiler.org) with the spot detection pipeline.

### Analysis of Cell Morphology

NHEKs cultured in 96-well plate to 60% confluence were stained with 100 nM MitoTracker® Red CMXRos (Thermo Fisher Scientific) and 5 μg/ml Hoechst 33342. BP IgGs (2 mg/ml) or normal IgG (2 mg/ml) were then added to the culture, which was immediately imaged. Nuclear masks were generated from images of Hoechst 33342 staining at 0 and 2 h, and the found edge and overlay functions of ImageJ software were employed to identify enlarged cell nuclei. The cytoplasm was visualized by overlaying the images of MitoTracker® Red CMXRos staining with nuclear staining. The whole-cell area and the area of typical macropinosomes was determined in phase-contrast images, and the numbers of total observed cells and the cells that formed macropinosomes were counted using ImageJ software (imagej.nih.gov/ij/). For the cell size calculation, nuclear staining was used to mark each individual cell, and then phase-contrast images matched to nuclear channel images were employed to calculate the size of each cell using In Cell Analyzer 1000 Workstation software with the multitarget analysis module. The cell size was calculated from the sum of pixel areas for each cell based on the pixel sizes of the images (2.73 pixels/μm).

NHEKs were treated with BP-1 IgG (2 mg/ml) or normal IgG (2 mg/ml) and imaged to identify the rupture of the plasma membrane. Bright field images of cells were obtained at 10 min intervals up to 16 h. The found edge and overlay functions of ImageJ software were employed to analyze the images.

### MitoTracker® Red CMXRos Staining Assay

NHEKs cultured in 96-well plates to 60% confluence were labeled with MitoTracker® Red CMXRos and Hoechst 33342 and treated with BP IgGs (2 mg/ml) or normal IgG (2 mg/ml) for 16 h to calculate the relative signal intensities and numbers of MitoTracker® Red CMXRos fluorescent dots per cell. Cells were fixed with cold 4% (w/v) paraformaldehyde for 10 min, and images of the cells were captured using an In Cell analyzer 2000 equipped with a ×20 objective (GE Biosciences). Images were quantified using CellProfiler image analysis software with the spot detection pipeline.

### Cellular Reactive Oxygen Species (ROS) Assay

Intracellular ROS production in NHEKs was measured using the fluorescent dye 2′,7′-dichlorofluorescein diacetate (DCFHDA, Abcam) according to the manufacturer's instructions. Cells cultured in a 96-well plate to 60% confluence were incubated with DCFHDA for 45 min at 37°C in the dark, and fresh medium containing BP IgGs (2 mg/ml) or normal IgG (2 mg/ml) were added and cultured for 6 h at 37°C. The fluorescence intensity was determined using a fluorescence microplate reader (DTX880, Beckman Coulter, Brea, CA, USA). The relative ROS fluorescence intensity was calculated as follows: [T_6h_ (fluorescence intensity at 6 h)–T_0h_ (fluorescence intensity at 0 h)]/numbers of adherent cells measured by cell counting.

### Mitochondrial Membrane Potential Assay

The mitochondrial membrane potential was measured using a JC-1 mitochondrial membrane potential assay kit (Mitosciences, Abcam) according to the manufacturer's instructions. NHEKs cultured in a 96-well culture plate to 60% confluence were stained with the JC-1 dye and incubated with BP IgGs (2 mg/ml) or normal IgG (2 mg/ml) for 20 h. The fluorescence intensity was analyzed using a fluorescence microplate reader (DTX880).

### Analysis of C_12_-Resazurin/SYTOX Green Staining

A C_12_-resazurin/SYTOX green kit (Molecular Probes, Eugene, OR, USA) was used to visualize the cell viability according to the manufacturer's instructions. Briefly, NHEKs cultured to 60% confluence in T-25 flasks in the presence or absence of inhibitors were incubated with BP IgGs (2 mg/ml) or normal IgG (2 mg/ml) for 16 h. Cells were harvested with the TrypLE Express Enzyme (Gibco) and resuspended in 100 μl of DPBS containing 1 μM SYTOX green and 50 nM C_12_-resazurin. After a 15 min incubation in the dark, 400 μL of DPBS were added. The cell suspension was mixed gently and then immediately subjected to flow cytometry analysis.

### Cell Motility Assay

NHEKs were incubated in 96-well culture plates (Falcon) to ~60% confluence. Cells cultured in the presence or absence of inhibitors were labeled with Hoechst 33342, and then treated with BP IgGs (2 mg/ml) or normal IgG (2 mg/ml). Images of the cells were captured at 10 min intervals up to 6 h using an In Cell analyzer 2000 equipped with a ×20 objective (GE Biosciences). The motility of ~2,000 cells was analyzed using ImageJ software with WrMtrack plugins (www.phage.dk/plugins/wrmtrck.html). The Euclidean distance and velocity were calculated using the Chemotaxis and Migration Tool (Ibidi GmbH) and the mean square displacements were obtained using Motility Lab (www.motilitylab.net/).

### Statistical Analysis

The results are presented either as the mean values ± standard errors of the means or mean values ± standard deviations (*SD*) from at least triplicate experiments. One-way or two-way ANOVA or Student's *t*-test was used to compare the means, and differences were deemed significant when the calculated *p*-value was < 0.05. All statistical analyses were performed with the R language (www.r-project.org).

## Results

### Kinetics of the Cell Surface IgG Binding, ColXVII Expression, and the Numbers of Adherent NHEKs Treated With BP IgG

**Figure 1:**
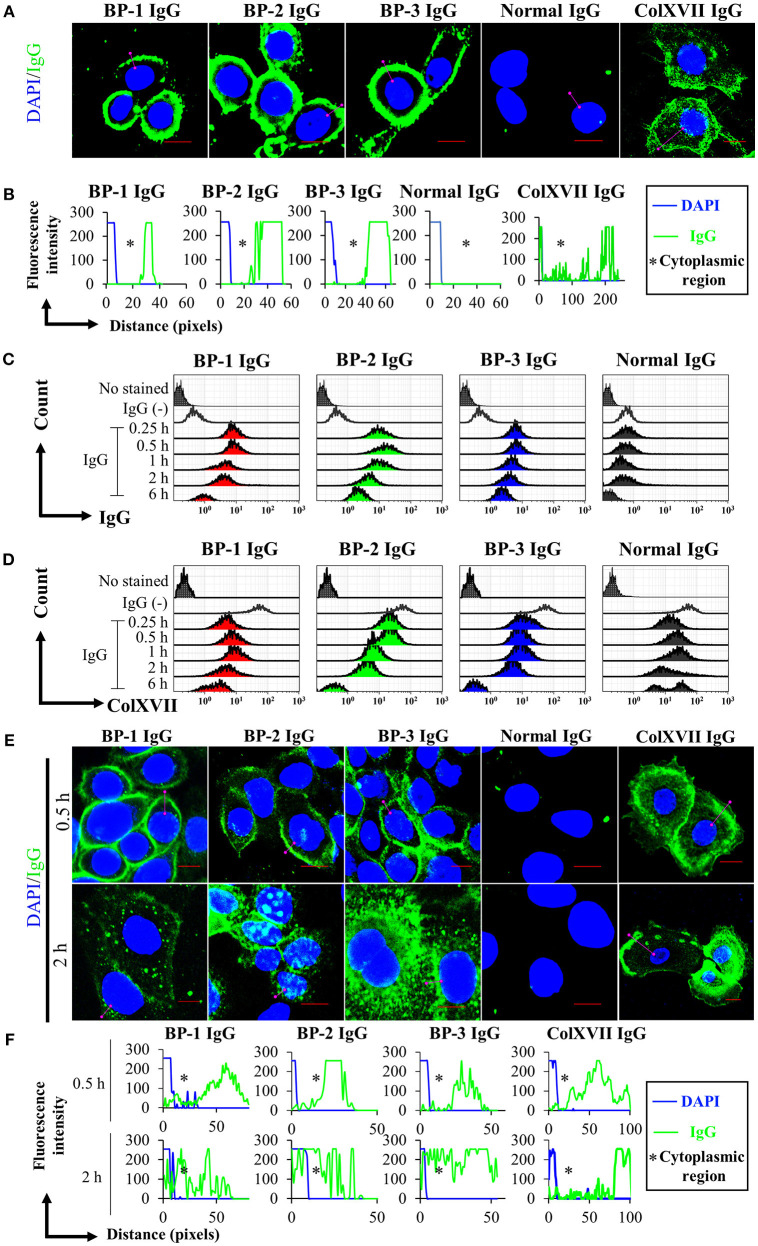
Kinetics of the cell surface IgG binding and ColXVII expression. **(A)** Immunofluorescence staining for IgG bound to fixed NHEKs. NHEKs were fixed with paraformaldehyde and permeabilized. Cells were incubated with BP IgGs (BP-1, BP-2, or BP-3, 2 mg/ml), normal IgG (2 mg/ml), or rabbit anti-human ColXVII COOH IgG (ColXVII IgG, 12 μg/ml) and then subsequently stained with FITC- or Alexa Fluor 488-conjugated secondary antibodies (green). The nucleus was stained with DAPI (blue). Scale bar 10 μm. **(B)** Quantification of the fluorescence intensity of IgG bound to fixed NHEKs. Data were analyzed using ICY software. **(C)** Evaluation of the cell surface IgG binding using flow cytometry. NHEKs were cocultured with 2 mg/ml IgGs for the indicated times and then immediately fixed without permeabilization. The cells were directly incubated with anti-human IgG-FITC and were examined using flow cytometry. **(D)** Evaluation of the cell surface ColXVII expression using flow cytometry. NHEKs were cocultured with 2 mg/ml IgGs for the indicated times, and then immediately fixed without permeabilization. Then, cells were first incubated with an IgG specific for human ColXVII-COOH, and then incubated with Alexa Fluor 488-conjugated anti-IgG. Cells were examined using flow cytometry. **(E)** Fluorescence microscopy images of the binding of the indicated IgGs to NHEKs. NHEKs were incubated with BP IgGs (BP-1, BP-2, or BP-3, 2 mg/ml), normal IgG (2 mg/ml), or ColXVII IgG (12 μg/ml) for the indicated times. Then, the cells were fixed, permeabilized, subsequently stained with FITC- or Alexa Fluor 488-conjugated secondary antibodies (green). The nucleus was stained with DAPI (blue). Scale bar 10 μm. **(F)** Quantification of the fluorescence intensity of IgG bound to NHEKs. Data were analyzed using ICY software.

**Figure 2:**
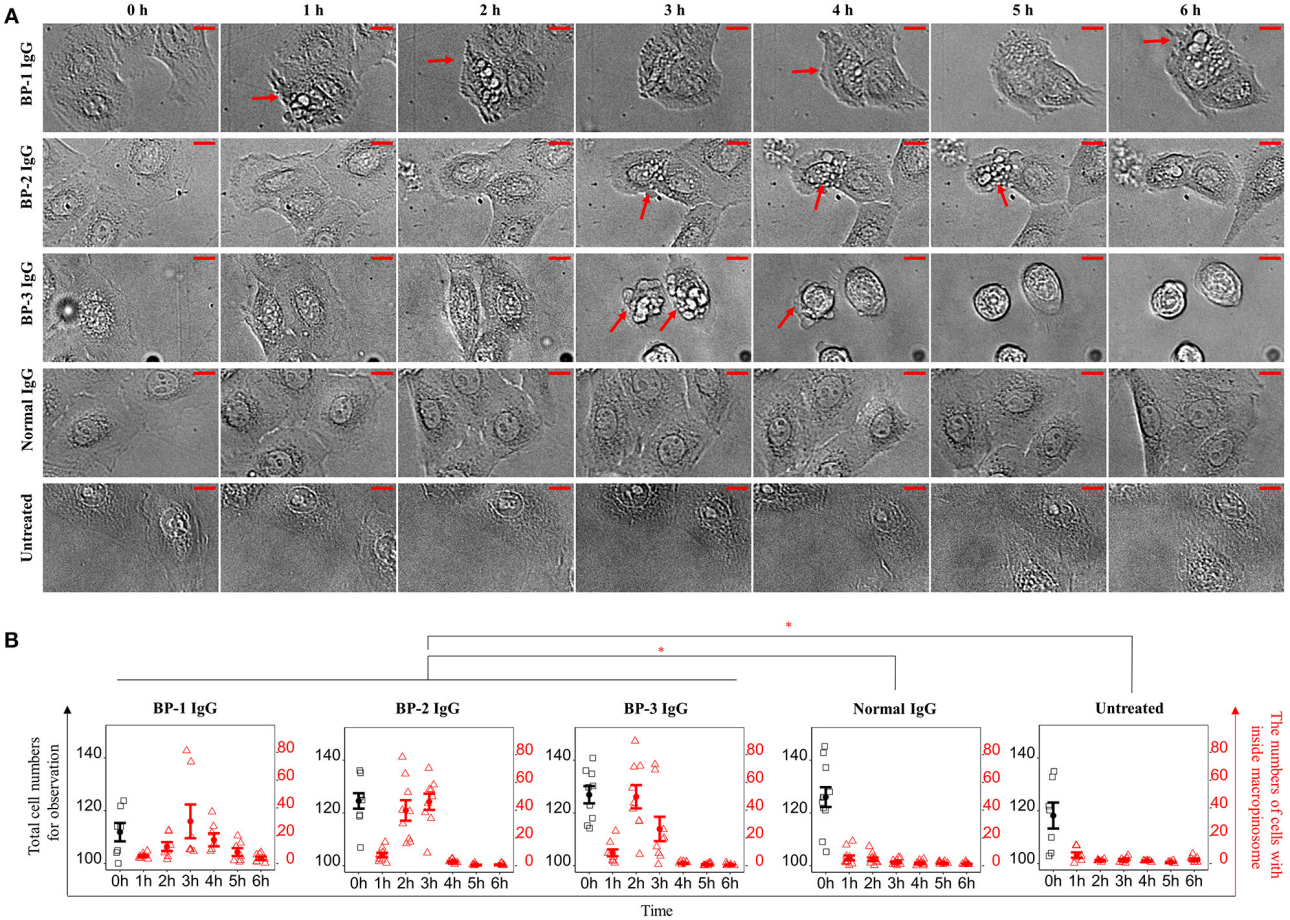
Phase-contrast images of macropinosome formation in NHEKs treated with BP IgG. **(A)** Phase-contrast images were captured every 10min after the addition of IgGs obtained from three patients with BP (BP-1, BP-2, and BP-3) or normal IgG to NHEKs and culture for up to 6 h. The figure shows images captured every 1 h. Cells with macropinosomes were indicated with arrows. Scale bar 10 μm. **(B)** The numbers of cells present during BP IgGs stimulation. Black: Total cell numbers observed at 0 h. Red: Numbers of cells containing macropinosomes at the indicated time points. Two-way ANOVA was used to analyze the significance of differences induced by BP IgGs. *p-value < 0.05.

We performed immunostaining and analyzed the results with a confocal laser-scanning microscope and flow cytometry to obtain spatial and kinetic information about BP IgG binding and ColXVII expression in NHEKs treated with BP IgGs. BP IgGs binding to the NHEK surface were verified by positive immunostaining for FITC-labeled anti-human IgG, and ColXVII expression on the surface of NHEKs was verified using ColXVII IgG (Figures 1A,B). In the flow cytometry analysis, the cell surface IgG binding and ColXVII expression on the surface of BP IgG-stimulated NHEKs decreased over time, whereas ColXVII expression and the binding of IgG to normal IgG-stimulated cells was not obviously altered (Figures 1C,D). Based on the confocal laser-scanning microscope observations, the cell surface localization of BP IgGs were visualized after a 0.5 h incubation, and most of the punctate IgGs staining were mainly localized in the cytoplasmic area at 2 h (Figures 1E,F). No significant IgG staining was observed when NHEKs were cocultured with normal IgG (Figures 1A–E). Immunostaining with ColXVII IgG did not show obvious internalization (Figures 1A,B,E,F). Phase-contrast images of NHEKs cocultured with BP IgGs displayed the BP IgG-induced formation of typical macropinosomes in NHEKs (Figure 2A), and compared to normal IgG- or untreated cells, BP IgG-stimulated cells exhibited a significant increase in the number of cells containing macropinosomes (Figure 2B).

**Figure 3:**
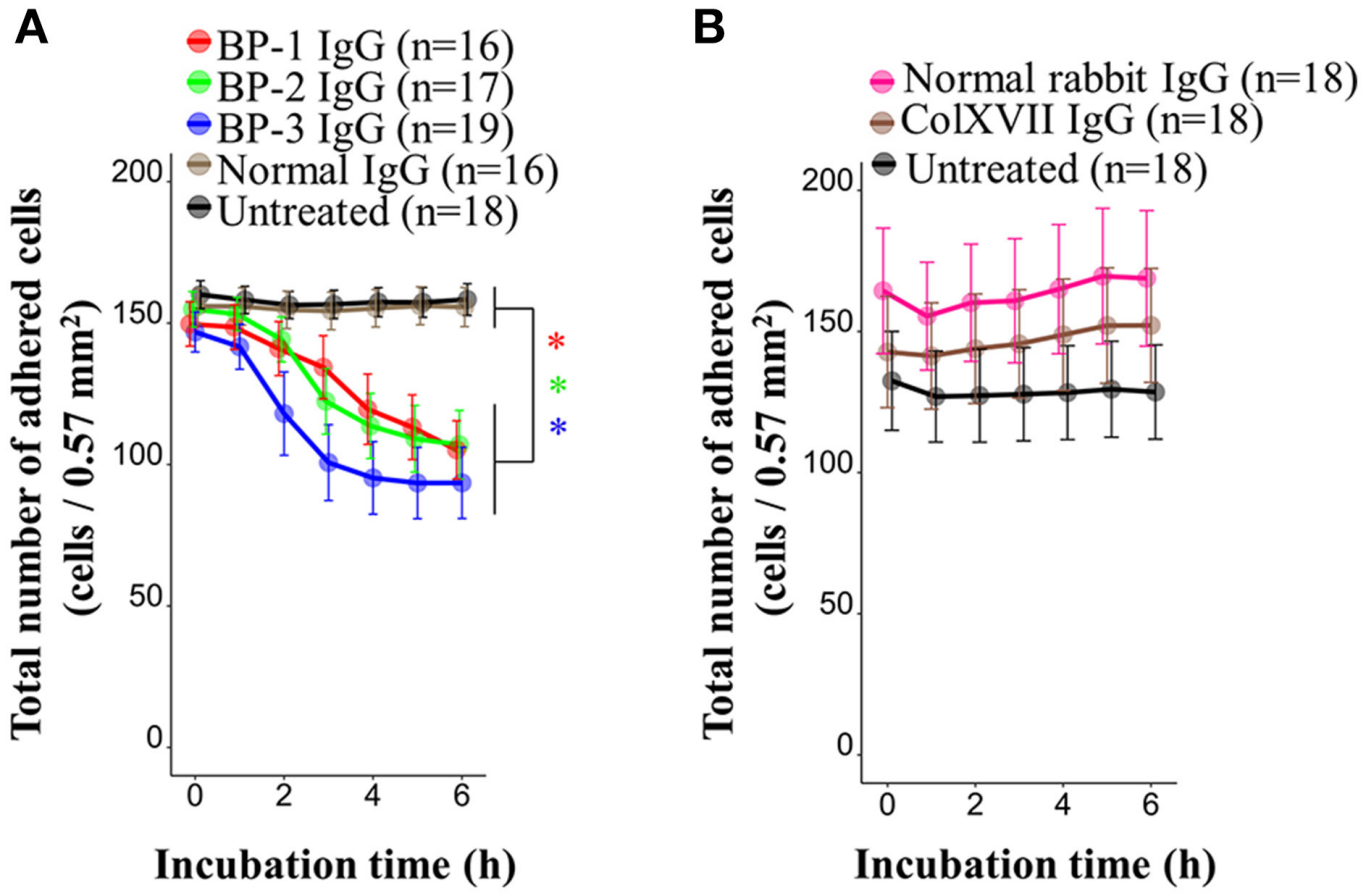
Kinetics of the numbers of adherent NHEKs stimulated with BP IgG. Total numbers of adherent cells after stimulation with **(A)** BP IgGs, normal IgG, or **(B)** ColXVII IgG or normal rabbit IgG. The cell numbers were counted hourly in a ×20 area (0.57 mm^2^) for up to 6 h. Two-way ANOVA was used to determine the significance of differences induced by BP IgGs. *p-value < 0.05.

BP IgG is known to cause the detachment of keratinocytes in 40% confluent cultures, although the adhesive function of ColXVII remains unclear (37). We performed a cell counting assay with living cells to determine the effect of BP IgGs on keratinocyte adhesion. BP IgGs significantly decreased the number of attached cells (reduced by ~40%) compared to the cells treated with normal IgG or untreated cells, and the effects were evident beginning 2 h after the incubation (Figure 3A). We also assessed the effect of IgG on detaching the COOH-termini of ColXVII (ColXVII IgG) and found that the addition of ColXVII IgG to the NHEKs did not obviously affect adhesion (Figure 3B).

### BP IgG Treatment Alters the Morphology of NHEKs

**Figure 4:**
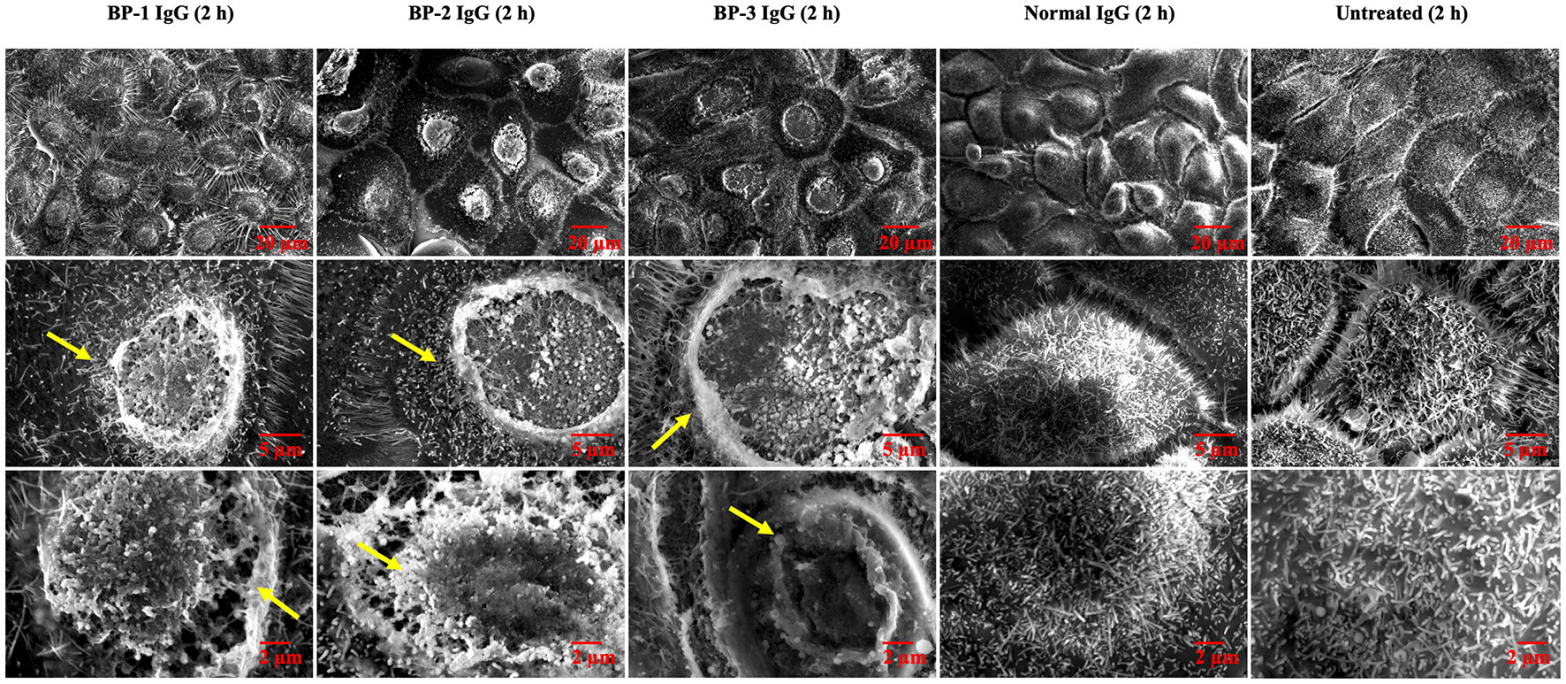
SEM images of NHEKs stimulated with BP IgG. SEM images of NHEKs treated with BP IgGs (BP-1, BP-2, and BP-3) or normal IgG for 2 h. The lower magnification pictures show the connections of cells and filopodia. The higher magnification pictures show the cell surface and microvilli of individual cells. The alterations in the cell membrane structure are indicated with arrows.

We hypothesized that internalization of BP IgG-ColXVII complexes alters the properties and morphology of NHEKs. First, we used SEM to characterize the status of the plasma membrane after BP IgGs were added to the culture. Compared to the cells incubated with normal IgG and/or the untreated cells, the BP IgG-stimulated cells exhibited alterations in the cell membrane structure (indicated with arrows in Figure 4).

**Figure 5:**
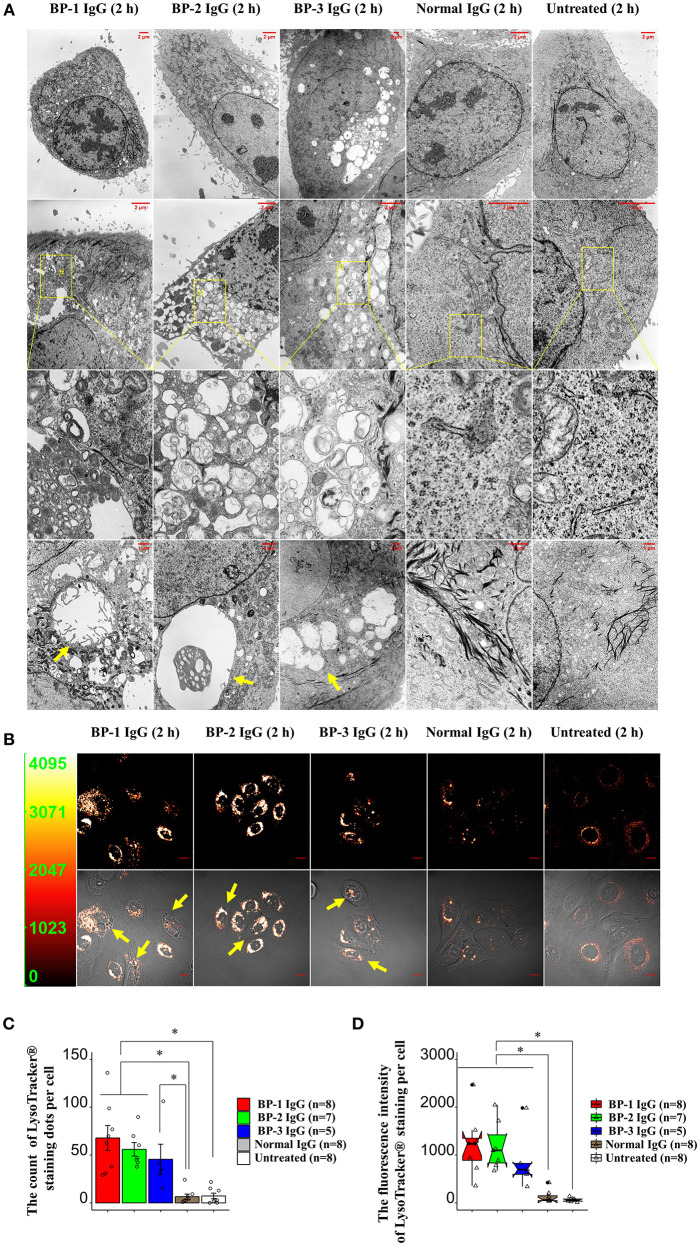
Changes in the morphology of NHEKs stimulated with BP IgG. **(A)** TEM images of NHEKs stimulated with BP IgGs (BP-1, BP-2, and BP-3) or normal IgG for 2 h. Zoomed-out images labeled with the # symbol shows vacuolar structures containing myelin-like structures. Vacuolar structures are indicated with arrows. The intracellular distribution of lysosomes in NHEKs stimulated with BP IgGs (BP-1, BP-2, and BP-3) or normal IgG. After a 2 h incubation with IgGs, cells were incubated with 50 nM LysoTracker® Green DND-26 for 15 min in dark, and live cells were imaged using confocal microscopy. Arrows indicate vesicles surrounded by lysosomes. Scale bar 10 μm. Y-axis in the left panel: fluorescence intensity. **(C)** Quantification of the fluorescently stained dots in cells. The LysoTracker® Green DND-26-stained dots in the cytoplasmic region of individual cells were counted using the CellProfiler image analysis software. One-way ANOVA was used for the statistical analysis. *p-value < 0.05. **(D)** Measurement of the fluorescence intensity per cell. The fluorescence intensity of LysoTracker® Green DND-26-stained cells was measured using CellProfiler image analysis software. One-way ANOVA was used for the statistical analysis. *p-value < 0.05.

The TEM examination revealed larger vesicles with villi-like structures inside the cytoplasmic space in BP IgG-treated NHEKs. BP IgG-treated NHEKs displayed vacuolar structures containing myelin-like structures. BP IgG-stimulated cells accumulated lysosomes and/or autophagosomes (Figures 5A,B), whereas cell nuclei showed neither damage nor chromatin condensation (Figure 5A). Since lysosomes have been suggested to play important roles in cell death (50), we further characterized the involvement of lysosomes in the BP IgG-induced cell death using a LysoTracker® Green DND-26 uptake assay. The live cell imaging revealed the accumulation of lysosomes 2 h after BP IgGs were added to the culture (Figure 5B), and the intracellular distribution of lysosomes was consistent with the TEM observations. Moreover, BP IgG-stimulated cells contained a greater number intracellular LysoTracker® Green DND-26-labeled dots with high fluorescent intensity (Figures 5C,D).

### Morphological and Functional Changes in the Mitochondria of NHEKs Incubated With BP IgG

**Figure 6:**
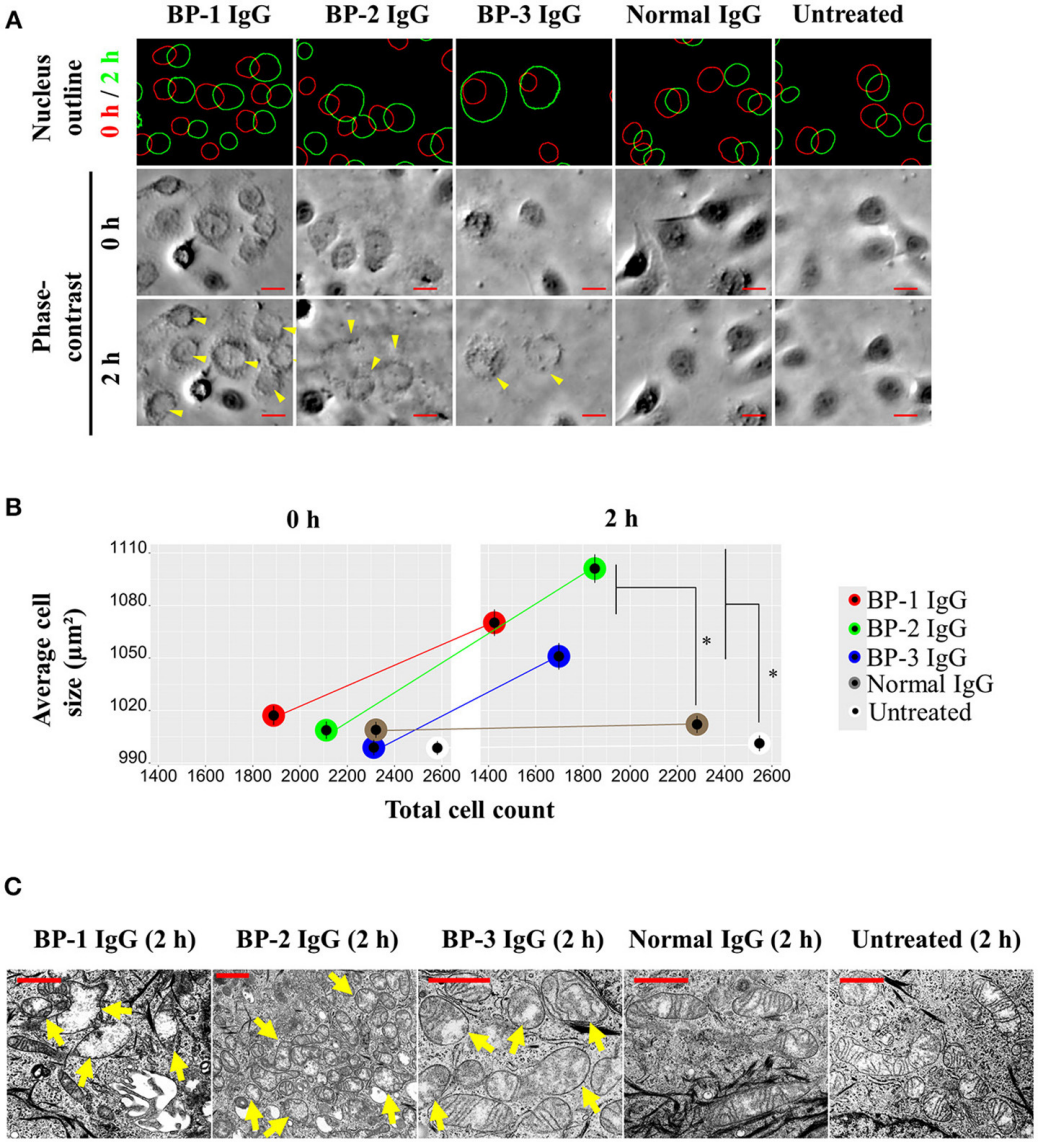
Changes in the mitochondrial morphology in NHEKs treated with BP IgG. NHEKs were stained with Hoechst 33342 and then stimulated with BP IgGs (BP-1, BP-2, and BP-3) or normal IgG. **(A)** Nuclei were labeled at 0 h (red circle) and 2 h (green circle); the whole-cell area is shown with a phase-contrast image. Arrows indicate enlarged cells. Scale bar 20 μm. **(B)** Average cell sizes at 0 and 2 h were calculated from at least 1,500 individual cells per group. Two-way ANOVA was used for the statistical analysis. *p-value < 0.05. **(C)** TEM images of mitochondrial morphology in NHEKs that had been incubated with the indicated IgGs for 2 h. Damaged mitochondria are indicated with arrows. Scale bar 1 μm.

**Figure 7:**
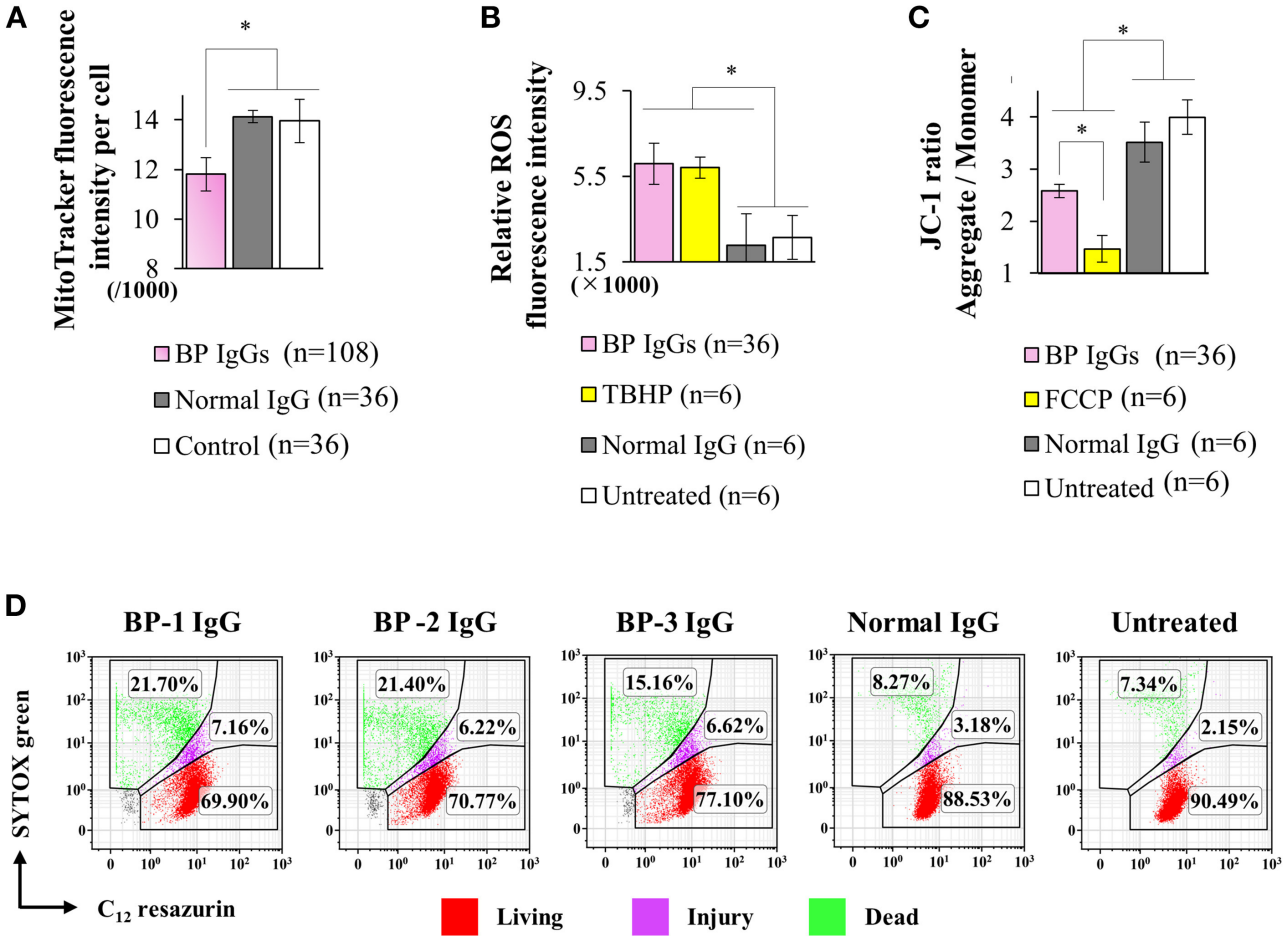
Effects of BP IgG on mitochondrial function in NHEKs. **(A)** Mitochondrial staining intensities (MitoTracker® Red CMXRos) were evaluated in NHEKs incubated with a pool of BP IgGs (from BP-1, BP-2, and BP-3) or normal IgG for 16 h. One-way ANOVA was used for the statistical analysis. *p-value < 0.05. **(B)** ROS were detected in NHEKs stimulated with BP IgGs or normal IgG for 6 h. TBHP was used as a positive control. One-way ANOVA was used for the statistical analysis. *p-value < 0.05. **(C)** The mitochondrial membrane potential was assayed in NHEKs treated with BP IgGs or normal IgG for 20 h. FCCP was used as a positive control. One-way ANOVA was used for the statistical analysis. *p-value < 0.05. **(D)** NHEKs were collected after a 16 h incubation with the indicated IgGs, then stained with SYTOX green (stains dead cells) and C_12_-resazurin (stains living cells), and subjected to flow cytometry.

We used time-lapse microscopy to analyze cell morphology and calculated the sizes of individual cells to determine whether the increased number of intracellular vesicles increased the sizes of individual cells. The sizes of NHEKs increased 1 h after adding BP IgGs to the culture, followed by the rupture of the cell membrane (Supplementary Figure S1A). The larger cell sizes reverted to the average size of normal cells after 3 h (Supplementary Figure S1B). Following nuclear staining and the capture of phase-contrast images, the sizes of NHEKs that had been incubated with BP IgGs for 2 h increased (Figures 6A,B). The TEM observations of mitochondria revealed numerous swollen mitochondria and a loss of cristae in cells stimulated with BP IgGs (Figure 6C). The mitochondrial functions were further analyzed with MitoTracker® Red CMXRos staining to observe the effect of BP IgGs, and the mitochondrial numbers and staining intensity were quantified in each individual cell. After 16 h, the number of stained mitochondria per cell did not differ between BP IgG-treated cells and normal IgG-treated or untreated cells (data not shown), whereas the staining intensity of mitochondria in BP IgG-treated cells decreased (Figure 7A).

ROS were generated 6 h after BP IgGs were added to the culture, whereas normal IgG did not increase ROS levels compared to untreated cells (Figure 7B). By performing JC-1 staining to analyze the mitochondrial membrane potential (ΔΨm), JC-1 aggregates, a sign of intact mitochondria, were more abundant in the normal IgG-stimulated cells, and untreated cells. On the other hand, dysfunctional mitochondria with a dissipation of ΔΨm, as evidenced by the increased number of JC-1 monomers, were pronounced in BP IgG-treated NHEKs at 20 h (Figure 7C).

The two-color fluorescence assay that distinguishes metabolically active cells was performed using SYTOX green and C_12_-resazurin dye. SYTOX green labels cells with compromised plasma membranes, and C_12_-resazurin is used to assess mitochondrial metabolic activity. After a 2 h incubation with BP-1 IgG or normal IgG, the pattern of SYTOX green and C_12_-resorufin staining did not differ between BP-1 IgG-, normal IgG-stimulated, or untreated NHEKs (data not shown). Therefore, we postulated that BP IgG-induced ColXVII internalization does not immediately induce a loss of metabolic activity in NHEKs. Since the dissipation of ΔΨm is usually regarded as a sign of decreased cell viability (51) and ΔΨm was not significantly decreased in NHEKs incubated with BP IgGs for up to 6 h (data not shown), we harvested the NHEKs that had been incubated with BP IgGs for 16 h and performed SYTOX green/C_12_-resazurin staining. A greater percentage of BP IgG-stimulated cells was stained with SYTOX green and a relatively lower percentage was stained with C_12_-resazurin than normal IgG-stimulated or untreated cells (Figure 7D), suggesting a decrease in the metabolic ability of BP IgG-treated cells. As DNA fragments were not found (data not shown) and chromatin condensation was not observed in our TEM images of BP IgG-stimulated cells (Figure 5A), we concluded that BP IgG did not induce apoptosis.

### BP IgG Increased NHEK Motility

**Figure 8:**
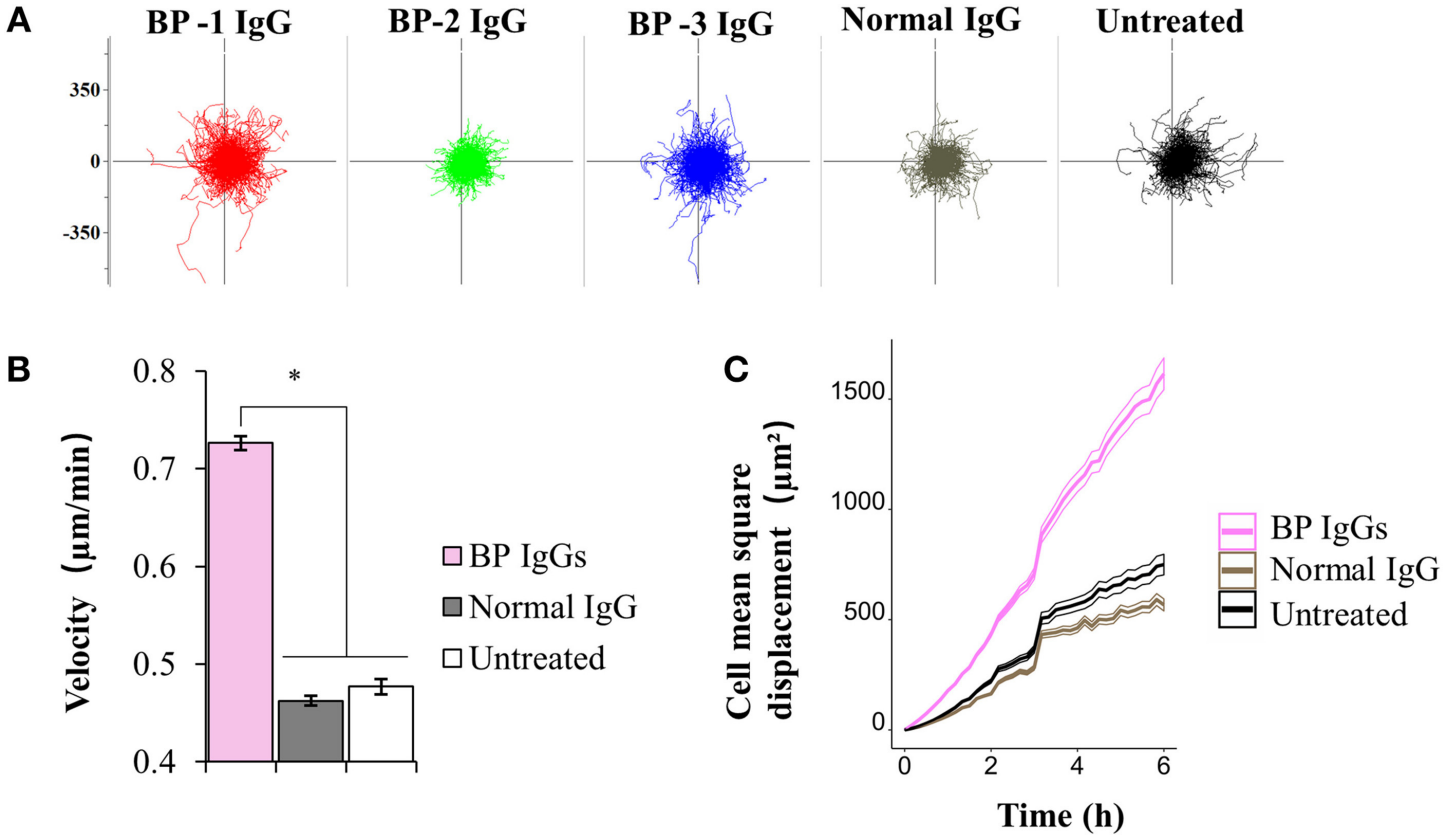
Results of the cell motility assay using NHEKs stimulated with BP IgG. Hoechst 33342-labeled NHEKs were incubated with IgGs obtained from 3 patients with BP (BP-1, BP-2, and BP-3) or normal IgG, and imaged using time-lapse microscopy. Images were captured at 10min intervals for up to 6 h. **(A)** Migration displacement maps were generated by centering the cell migration paths at a common starting point. Axes of vector diagrams = 500 μm. **(B)** Cell velocity of NHEKs treated with IgGs. One-way ANOVA was employed to determine the significance of differences induced by BP IgGs. *p-value < 0.05. **(C)** Mean square displacement of the NHEKs treated with IgGs.

Next, we extended our observations to evaluate cell motility. According to the results of the electron microscopy analysis, BP-1 IgG-stimulated cells displayed longer lamellipodia (Figure 4), and the extension of lamellipodia is closely related to cell migration (52). Throughout the 6 h observation of live cells, BP IgG-treated cells showed increased cell motility compared with normal IgG-stimulated and untreated cells (Figure 8). Notably, both the migration distance (Figures 8A,C) and velocity (Figure 8B) increased.

### Effects of cytochalasin D, NSC237766, and MG132 on the BP IgG-Induced Alterations in keratinocytes

The findings of BP IgG-induced cell dysfunction led us to investigate the pathway involved in the BP IgG-induced alterations. Three inhibitors that have been established as involved in the ColXVII internalization process were employed to further investigate the direct effect of BP IgGs on cell adhesion, metabolic activity, and motility.

**Figure 9:**
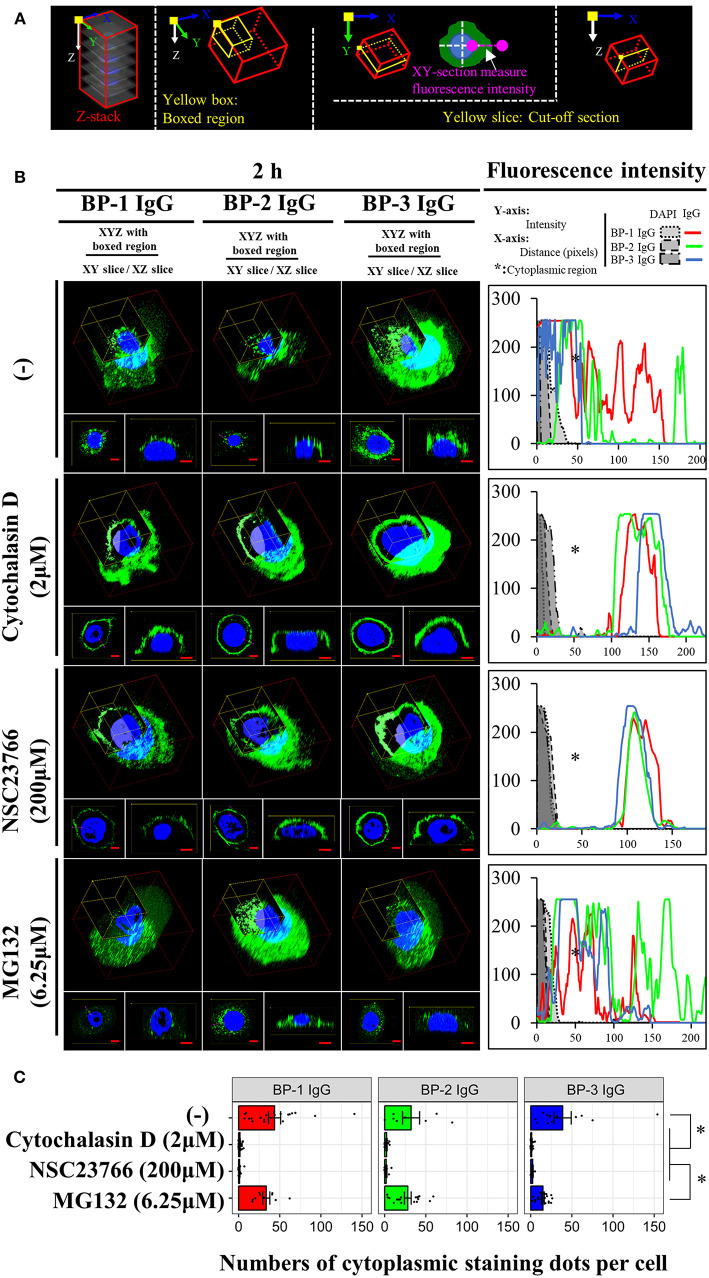
Effects of cytochalasin D, NSC237766, and MG132 on the BP IgG-induced internalization. **(A)** Overview of the 3D reconstruction assay. The 3D reconstruction was performed as described in the methods. The boxed region indicates XYZ boxed area, XY slice indicates the horizontal slice and used to measure the fluorescence intensity, and XZ slice indicates the longitudinal slice. **(B)** Fluorescence microscopy images of the binding of the indicated IgGs to the NHEKs at 2 h. NHEKs were pretreated with cytochalasin D (2 μM) for 30min, NSC23766 (200 μM) for 1 h, or MG132 (6.25 μM) for 0 h, and then 2 mg/ml BP IgGs (BP-1, BP-2, BP-3) or normal IgG were added to the cultured cells. The 3D reconstruction assay was applied to observe the changes. Scale bar 5 μm. **(C)** Quantification of the IgG-stained dots in cells. The anti-IgG-FITC-stained dots in the cytoplasmic region of individual cells were counted using the CellProfiler image analysis software. One-way ANOVA was used for the statistical analysis. *p-value < 0.05.

**Figure 10:**
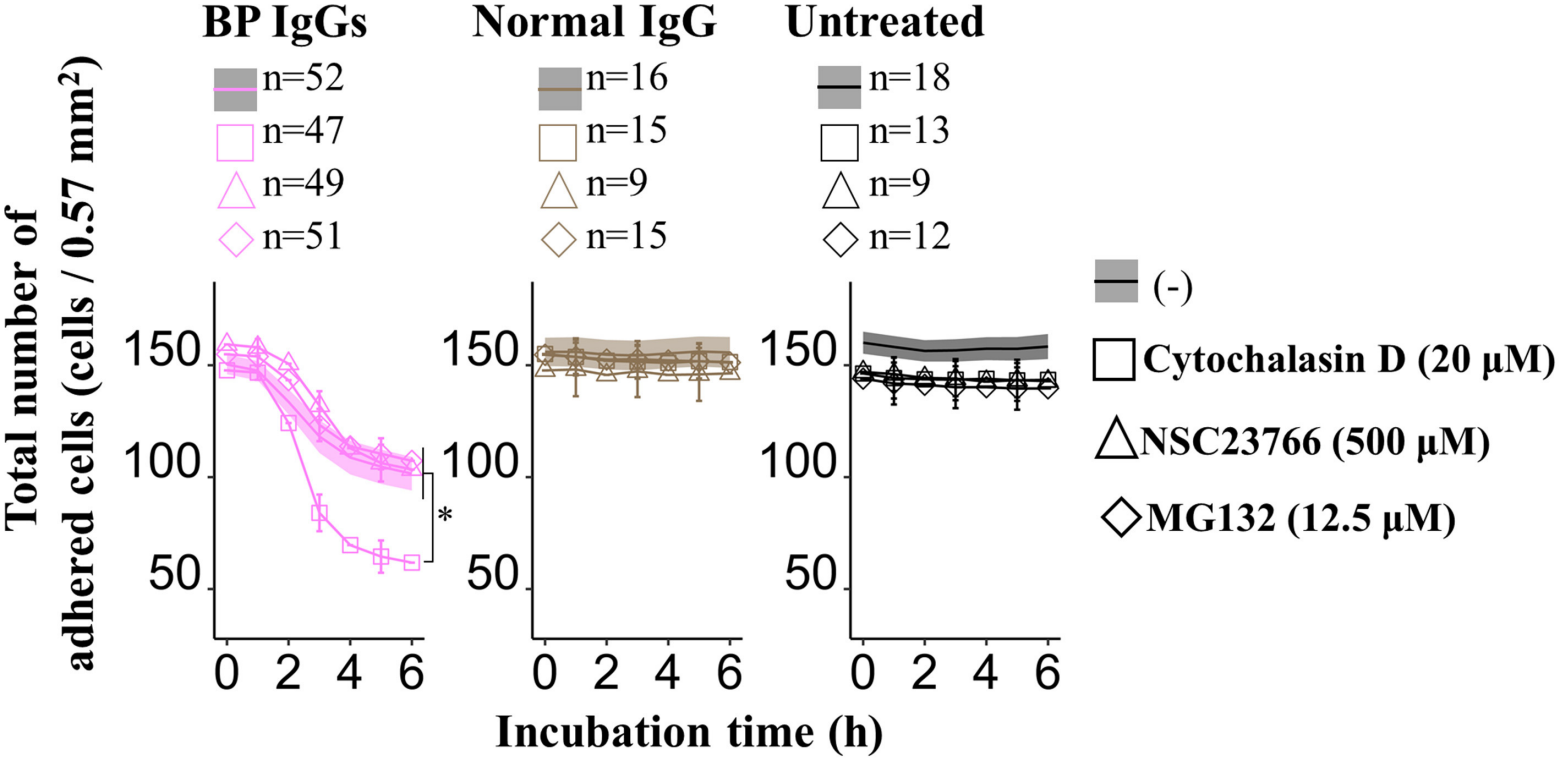
Effects of cytochalasin D, NSC237766, and MG132 on the BP IgG-induced decrease in the number of adherent cells. NHEKs were pretreated with cytochalasin D (20 μM) for 30min, NSC23766 (500 μM) for 1 h, or MG132 (12.5 μM) for 0 h, and then 2 mg/ml BP IgGs (BP-1, BP-2, and BP-3) or normal IgG were added to the cultured cells. Total numbers of adherent cells after the incubation with BP IgGs or normal IgG were counted every hour in a ×20 area (0.57 mm^2^) for up to 6 h. One-way ANOVA was used for the statistical analysis. *p-value < 0.05.

**Figure 11:**
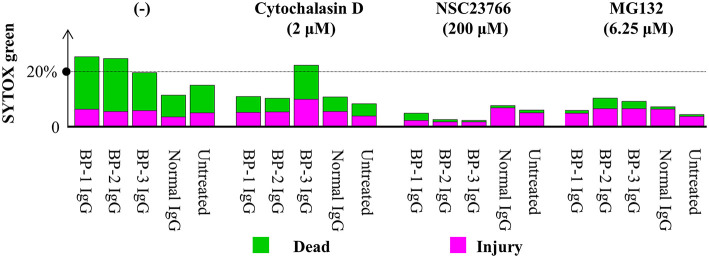
Effects of cytochalasin D, NSC237766, and MG132 on the BP IgG-induced decrease in cellular metabolic activity. NHEKs were pretreated with cytochalasin D (2 μM) for 30min, NSC23766 (200 μM) for 1 h, or MG132 (6.25 μM) for 0 h, and then 2 mg/ml BP IgGs (BP-1, BP-2, and BP-3) or normal IgG were added to the cultured cells for 16 h. Then, NHEKs were collected, stained with SYTOX green (stains dead cells) and C_12_-resazurin (stains living cells), and analyzed using flow cytometry.

**Figure 12:**
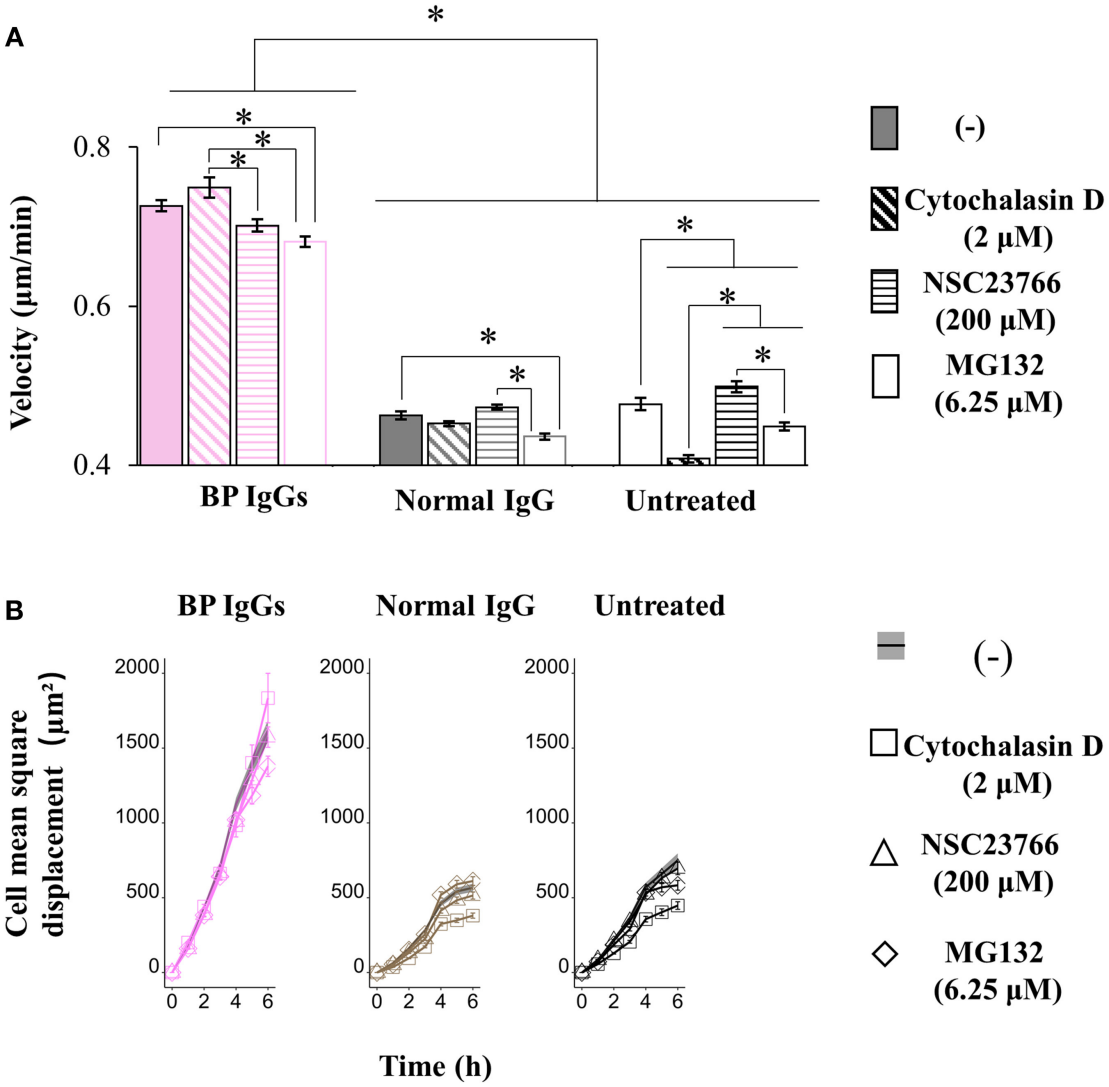
Effects of cytochalasin D, NSC237766, and MG132 on the BP IgG-induced increase in cell motility. NHEKs were pretreated with cytochalasin D (2 μM) for 30min, NSC23766 (200 μM) for 1 h, or MG132 (6.25 μM) for 0 h, and then 2 mg/ml BP IgGs (BP-1, BP-2, BP-3) or normal IgG were incubated with the cultured cells for 6h. **(A)** Velocity of the NHEKs stimulated with IgGs. One-way ANOVA was used for the statistical analysis. *: *p*-value < 0.05. **(B)** Mean square displacement of the NHEKs stimulated with IgGs.

Consistent with previous reports (43), application of cytochalasin D (inhibitor of the actin-cofilin interaction) inhibited the internalization of the BP IgG-ColXVII immune complex in BP IgG-stimulated NHEKs (Figures 9A–C). In the cell counting assay, the application of cytochalasin D did not exert a protective effect but instead increased the adhesion of NHEKs treated with BP IgGs (Figure 10). The cytochalasin D treatment did not completely relieve the BP IgG-induced loss of metabolic activity in NHEKs (Figure 11). In the motility assay, cytochalasin D significantly decreased the migration speed and distance of untreated NHEKs (Figure 12A), although it did not consistently exert the inhibitory effect on the NHEKs stimulated with BP IgGs (Figures 12A,B).

MG132 (inhibitor for proteasome) suppresses ColXVII depletion in both mouse and human keratinocytes stimulated with BP IgG (41). Thus, we hypothesized that the reduction in the adhesion of NHEKs treated with BP IgG is related to the depletion of ColXVII. MG132 did not prevent BP IgG-induced internalization (Figures 9A–C) or delay the BP IgG-induced decrease in cell number (Figure 10). However, MG132 protected NHEKs from the BP IgG-induced loss of metabolic activity (Figure 11). These data support our hypothesis that the metabolic activity was associated with proteasome activation in NHEKs stimulated with BP IgGs. Additionally, MG132 slightly decreased the migration of BP IgG-treated cells (Figures 12A,B).

The formation of macropinosomes in epithelial cells as an early response to combat intracellular pathogens requires the activation of a Rho-GTPase such as Rac1 (46, 53). NSC23766 (a Rac1 inhibitor) suppresses the internalization of BP IgG-ColXVII immune complexes (54). In the immunostaining assay, we also confirmed that the inhibition of Rac1 activity prevented BP IgG-ColXVII internalization (Figures 9A–C). Surprisingly, the application of NSC23766 to the NHEKs treated with BP IgGs did not exert a protective effect on adhesion (Figure 10). As shown in Figure 11, the application of NSC23766 to the BP IgG-treated NHEKs prevented the loss of metabolic activity, but did not reduce the cell motility (Figure 12).

## Discussion

As shown in the present study, BP IgG directly induces NHEK dysfunction, as BP IgG stimulation increases SYTOX green staining, revealing structural disintegration of the plasma membrane, intercellular vesicle formation, and mitochondrial dysfunction, but not chromatin condensation. In addition, BP IgG induced cell dysfunction by activating Rac1 and the proteasome. Our findings support the hypothesis proposed by Ujiie et al. that BP IgG directly causes subepidermal bulla formation in patients with BP (41).

We considered two possible explanations for BP IgG-induced cell dysfunction: methuosis or oncosis. The well-known response of NHEKs to BP IgG stimulation is macropinosome formation (43, 54). In mammalian cells, hyperstimulation of macropinosomes is known to lead to methuosis, which is defined as the maturation of macropinosomes (48, 49). We first tested whether the BP IgG-induced accumulation of fluid-filled endocytic vesicles was catastrophic and ultimately merged with the lysosomes, as previously reported (55), to distinguish between these mechanisms. However, refuting our methuosis hypothesis, BP IgG-induced macropinosomes did not merge with lysosomes, excluding the possibility of methuosis. The alternative explanation is oncosis, a type of cell dysfunction caused by cell membrane damage characterized by cellular and/or organelle swelling (56). Our ultrastructural and morphological observations were consistent with these changes (Figures 4, 5 and Supplementary Figure S1).

Importantly, our data provided evidence supporting the relationship between BP IgG-induced cell dysfunction and lysosome accumulation and mitochondria dysfunction (Figures 5–7). Cell membrane injury directly results in acute cell dysfunction, and the dying cells release endogenous alarm signals, which trigger the innate immune system and modulate inflammation through the lysosome-induced increase in the levels and excessive formation of ROS (50, 57). In contrast, the lysosomes mediate plasma membrane repair and control cellular dysfunction (50, 58). Mitochondrial dysfunction usually leads to mammalian cell death and influences the immune system to perceive/react to the dying cells (51, 59). Lysosome-mediated cellular dysfunction is characterized by simultaneous membrane damage and the proteolysis of a wide range of proteins (60). Lysotracker® Green DND-26, a marker of the lysosome membrane potential (60), helped identify BP IgG-treated cells with a higher lysosome membrane potential. Conceivably, the proteolytic enzymes released from lysosomes trigger a forward loop promoting lysosome rupture after a 2 h treatment with BP IgG. Moreover, the proteasome inhibitor MG132 efficiently blocks lysosome rupture (61) and has attracted considerable attention as an anti-BP agent (41). Therefore, MG132 likely exerted its protective effects on BP IgG-induced cell dysfunction in our study through its antilysosome/proteasome effects. Not surprisingly, several skin diseases are reported to be associated with alterations in mitochondria-related metabolic pathways (59). Consistent with the results of our ROS assay, the BP disease process was recently reported to be characterized by ROS production (62). We provided direct evidence that cell membrane fragility follows ROS production induced by BP IgG stimulation, which ultimately significantly increases cell membrane fragility, although we have not identified the molecular events underlying ROS production. The cell membrane is the site of activation of numerous signaling cascades that induce mitochondrial dysfunction. Interestingly, Rac1 is reported to be located both at the plasma membrane and mitochondrial membrane (63). The administration of NSC23766 decreases ROS production and promotes cell survival (64). Rac1 activation might serve as the initial step to “destroy” both the cell and mitochondrial membranes, and the pathways downstream of Rac1 also contribute to increasing ROS production to promote cellular dysfunction.

We conclude that the persistent stimulation with BP IgG induced the formation of macropinosomes in keratinocytes, resulting in a fragile plasma membrane, intercellular vesicle formation, lysosome accumulation, and ROS production, which ultimately contribute to mitochondrial dysfunction. The BP IgG-induced alterations in keratinocytes trigger the immune system to digest the basal keratinocytes, causing the formation of blisters on the skin along with BMZ.

Our study has limitations. For example, we had limited explanations for the association of BP IgG-induced keratinocyte alterations with blister formation. Similar to the results from a previous study of basal keratinocytes (*in vivo*) from patients with BP (65), intercellular vesicles filled with villi and swelled mitochondria were also observed in our TEM experiment. We first considered that the intercellular vesicles likely form in response to the maturation of macropinosomes, and the lysosome-mediated pathway contributes to vesicle expansion and exerts potential effects on skin blistering. However, the intracytoplasmic accumulation of lysosomes/autophagosomes are not observed in ultrastructural images *in vivo* (65–67). In addition, histopathology rarely reveals degenerated keratinocytes in patients with early-stage BP. We considered that the pattern of ColXVII expression differs between keratinocytes *in vivo* (hemidesmosome ColXVII) and *in vitro* (nonhemidesmosome ColXVII) (15, 16, 21); future studies should provide insights into the different changes in keratinocytes *in vitro* and *in vivo* during the BP IgG-induced blistering process.

We also describe the role of ColXVII in regulating cell adhesion and motility (Figures 3, 8, 10, 12). The formation of the BP IgG-ColXVII complex has been shown to tear the weakened lamina lucida, leading to a specific split at the lamina lucida and induction of BMZ blistering (37). According to another report, ColXVII mediates the anchorage of basal keratinocytes by regulating cell motility (68). Thus, we speculate that the changes in the adhesion and motility of keratinocytes are involved in the pathogenesis of blistering in patients with BP. As shown in reports (69, 70), IgGs targeting proteins other than ColXVII-NC16a do not detach cells from culture dishes. Interestingly, an IgG targeting the C-terminus of ColXVII neither induced obvious IgG-ColXVII internalization nor had any significant effect on cell detachment. Together with the results of the *in vivo* study showing that IgGs targeting the ColXVII ectodomain fail to reproduce blistering in an animal model (71), the findings from previous studies and our data confirm the pathogenicity of the anti-ColXVII-NC16a antibodies in subjects with BP. Based on the existing literature, the reduction in the cell adhesion observed upon BP IgG stimulation can be accounted for by ColXVII internalization (43, 72). However, researchers have not clearly determined how ColXVII internalization might influence cell adhesion. In the present study, the BP IgG-induced cell detachment was not directly induced by macropinosome formation, because alterations in actin, the well-known and necessary molecule for macropinosome formation (73), did not completely prevent NHEK detachment. NHEKs disassembled their contacts with neighboring cells and detached from the culture dish following an incubation with BP IgG. Furthermore, epithelial cell “destabilization” has also been shown to require a step mediated by the proteasome (74). For this reason, we speculated and confirmed that the BP IgG-induced cell detachment was associated with proteasome activation, and the internalization of the IgG-ColXVII complex probably requires the initial event of proteasome activation. Another interesting aspect of this study was that the BP IgG treatment increased NHEK motility. Based on the BP IgG-induced cell detachment, we speculate that the BP IgG-induced alterations in cell motility are likely due to a decrease in the cell density. On the other hand, ColXVII has been shown to regulate keratinocyte motility, while changes in cell motility following the loss of ColXVII remain controversial (26). Studies using ColXVII-knockdown keratinocytes have reported that the loss of ColXVII reduces lamellipodial stability (75) and induces cell migration mediated by Rac1 (76, 77). Cell migration is associated with the remodeling of the actin cytoskeleton. However, cytochalasin D did not affect cell motility following the BP IgG treatment. This discrepancy might be explained by the binding of ColXVII to two different cytoskeleton systems in keratinocytes: actin-associated focal contacts and keratin-associated hemidesmosome compounds (15, 78, 79).

Our findings provide a better understanding of the direct effects of BP IgG on keratinocytes by increasing the fragility of the cell membrane, resulting in keratinocyte dysfunction, probably through oncosis. In addition, the BP IgG-induced cellular dysfunction was reversed by Rac1/proteasome inhibition. We believe that our identification of the Rac1/proteasome-mediated signaling pathway provides valuable new insights that have improved our understanding of the direct effects of BP IgG on keratinocytes.

## Author Contributions

DT designed the study and wrote the initial draft of the manuscript. XD contributed to data collection and interpretation, and critically reviewed the manuscript. KN contributed to data interpretation and critically reviewed the manuscript. NY and OY contributed to the electron microscopy experiments and data interpretation, and OY critically reviewed the manuscript. EM supervised the entire study, provided critical intellectual input, and approved the final version of the manuscript. All authors approved the final version of the manuscript and agree to be accountable for all aspects of the work and ensuring that questions related to the accuracy or integrity of any part of the work are appropriately investigated and resolved.

### Conflict of Interest Statement

The authors declare that the research was conducted in the absence of any commercial or financial relationships that could be construed as a potential conflict of interest.

## Supplementary Material

Supplementary Figure S1 Morphometric features and cell sizes. **(A)** NHEKs were cocultured with BP-1 IgG or normal IgG. Outlines of single cells at the indicated time points are shown. Arrows indicate the rupture of the plasma membrane. Scale bar 20 μm. **(B)** Analysis of the sizes of NHEKs incubated with IgG obtained from three patients with BP (BP-1, BP-2, and BP-3) or normal IgG. Images of nuclear staining were used to clearly segment the cells, and phase-contrast images were used to calculate cell sizes. Yellow dots indicate the average cell size. Approximately 1,500 cells from each group were analyzed. Student's *t*-test was used for the statistical analysis. ^*^*p*-value < 0.05.

## Abbreviations

BP: bullous pemphigoid
BMZ: basement membrane zone
BSA: bovine serum albumin
CLEIA: chemiluminescent enzyme immunoassay
ColXVII: type XVII collagen
DAPI, 4′: 6-diamidino-2-phenylindole
DCFHDA, 2′: 7′-dichlorofluorescein diacetate
DMSO: dimethyl sulfoxide
DPBS: dulbecco's phosphate-buffered saline
FCCP: carbonyl cyanide-4-(trifluoromethoxy)phenylhydrazone
GTPases: guanosine triphosphate phosphohydrolases
h: hour
IgG: immunoglobulin G
MG132: carbobenzoxy-Leu-Leu-leucinal
min: minutes
NC16a: noncollagenous 16a domain
NHEKs: normal human epidermal keratinocytes
NSC23766: n6-[2-(4-diethylamino-1-methyl-butylamino)-6-methyl-pyrimidin-4-yl]-2-methyl-quinoline-4,6-diamine trihydrochloride
Rac1: ras-related C3 botulinum toxin substrate 1
ROS: reactive oxygen species
RT: room temperature
SEM: scanning electron microscope
TBHP: tert-butyl hydroperoxide
TEM: transmission electron microscope
QY-1: n-butyl glycidyl ether
ΔΨm: mitochondria membrane potential.
