# Corrigendum: Bullous pemphigoid IgG induces cell dysfunction and enhances the motility of epidermal keratinocytes *via* Rac1/proteasome activation

**DOI:** 10.3389/fimmu.2022.1109597

**Published:** 2023-01-24

**Authors:** Duerna Tie, Xia Da, Ken Natsuga, Nanako Yamada, Osamu Yamamoto, Eishin Morita

**Affiliations:** ^1^ Department of Dermatology, Shimane University Faculty of Medicine, Izumo, Japan; ^2^ Department of Dermatology, Hokkaido University Graduate School of Medicine, Sapporo, Japan; ^3^ Division of Dermatology, Department of Medicine of Sensory and Motor Organs, Faculty of Medicine, Tottori University, Yonago, Japan

**Keywords:** bullous pemphigoid, keratinocyte, IgG, cell adhesion, cell migration

In the published article, there was an error in **Figure 4** as published. The [Untreated] and [normal IgG] groups had the same high-magnification pictures (Row 3, columns 3 and 4). There was a mistake with the [Untreated] group. The corrected **Figure 4** and its caption appear below.

**Figure 4:**
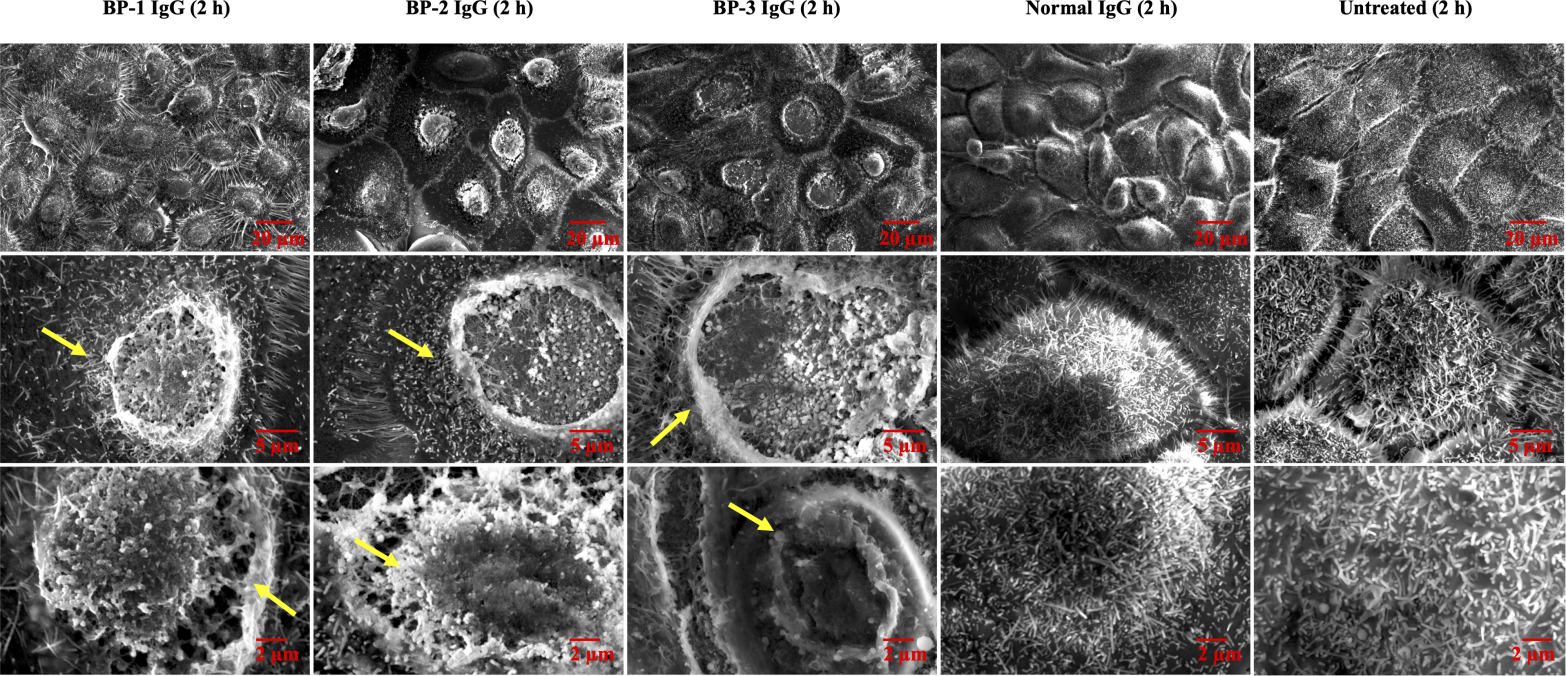
SEM images of NHEKs stimulated with BP IgG. SEM images of NHEKs treated with BP IgGs (BP-1, BP-2, and BP-3) or normal IgG for 2 h. The lower magnification pictures show the connections of cells and filopodia. The higher magnification pictures show the cell surface and microvilli of individual cells. The alterations in the cell membrane structure are indicated with arrows.

The authors apologize for this error and state that this does not change the scientific conclusions of the article in any way. The original article has been updated.

