# Supplementary figures and images for: Bullous Pemphigoid IgG Induces Cell Dysfunction and Enhances the Motility of Epidermal Keratinocytes via Rac1/Proteasome Activation
Source: Front Immunol. 2019 Feb 12;10:200. doi: 10.3389/fimmu.2019.00200 (PMC6379344; doi:10.3389/fimmu.2019.00200)

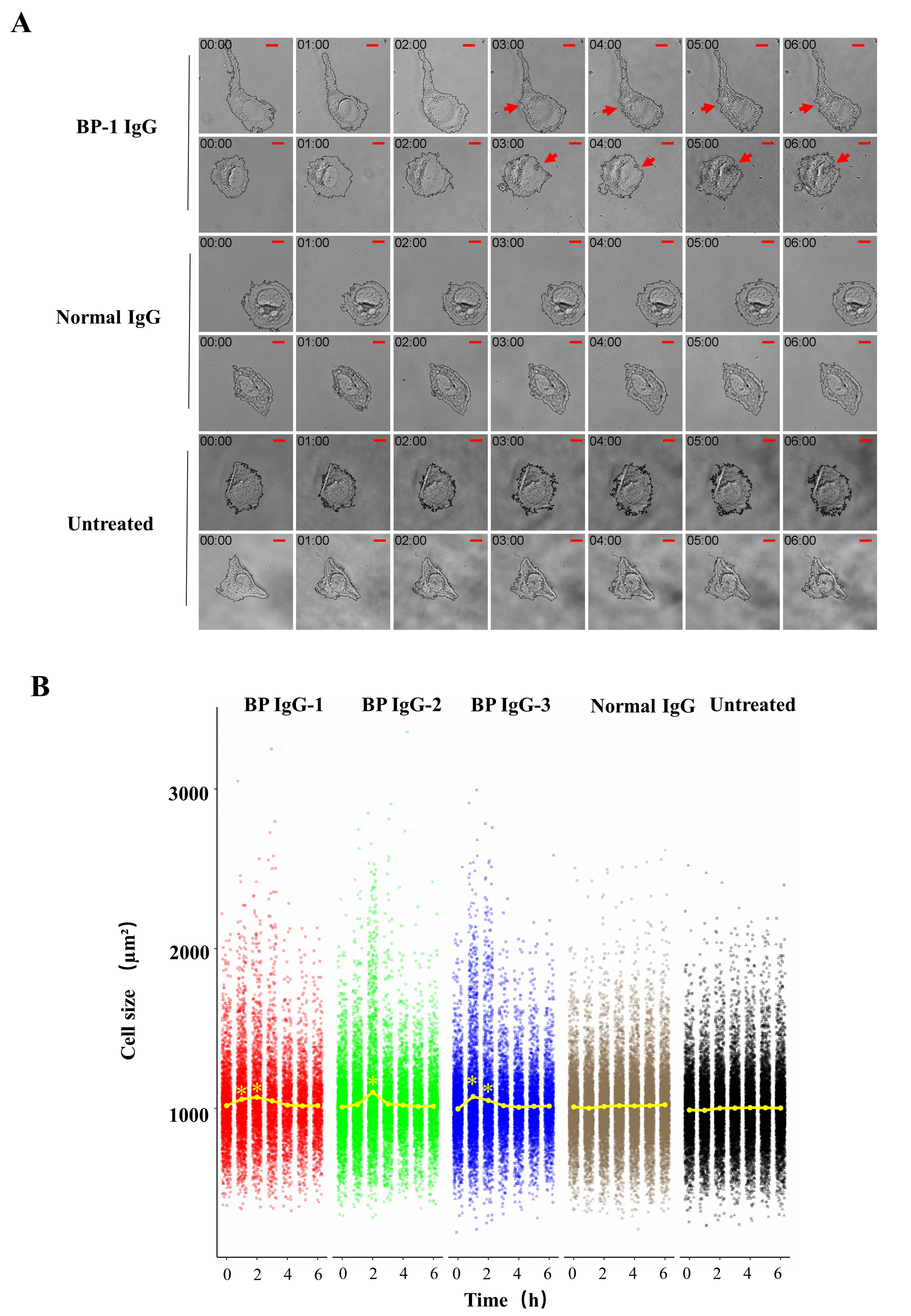

Supplement: Supplementary Figure S1 — Morphometric features and cell sizes. (A) NHEKs were cocultured with BP-1 IgG or normal IgG. Outlines of single cells at the indicated time points are shown. Arrows indicate the rupture of the plasma membrane. Scale bar 20 μm. (B) Analysis of the sizes of NHEKs incubated with IgG obtained from three patients with BP (BP-1, BP-2, and BP-3) or normal IgG. Images of nuclear staining were used to clearly segment the cells, and phase-contrast images were used to calculate cell sizes. Yellow dots indicate the average cell size. Approximately 1,500 cells from each group were analyzed. Student's t-test was used for the statistical analysis. *p-value < 0.05. [file Image_1.TIF]
